# Rapid Post-Recovery Changes in the Gill Holobiont of the Deep-Sea Mussel *Gigantidas haimaensis* During Short-Term Ex Situ Maintenance

**DOI:** 10.3390/biology15151305

**Published:** 2026-08-05

**Authors:** Gaoyou Yao, Yuyuan Wu, Mengfan Ren, Xusheng Guo, Hua Zhang, Haijun Tian, Hanzhi Xu, Maoxian He

**Affiliations:** 1 Fisheries College, Xinyang Agriculture and Forestry University, Xinyang 464000, China; 2 State Key Laboratory of Breeding Biotechnology and Sustainable Aquaculture, Guangdong Provincial Key Laboratory of Applied Marine Biology, Laboratory of Tropical Marine Bio-Resources and Ecology, South China Sea Institute of Oceanology, Chinese Academy of Sciences, Guangzhou 510301, China; 3 University of Chinese Academy of Sciences, Beijing 100049, China; 4 Yantai Marine Economy Research Institute, Yantai 264003, China

**Keywords:** *Gigantidas haimaensis*, deep sea, Haima cold seep, chemosymbiosis, gill microbiome, RNA-Seq, HSP70, dual oxidase 2, sulfide oxidoreductase, ribosome biogenesis

## Abstract

While chemosymbiosis offers deep-sea bathymodioline mussels a critical metabolic advantage, this dependence makes them vulnerable when removed from their native environment, and their early post-recovery dynamics remain poorly resolved. We maintained adult *Gigantidas haimaensis* collected from the Haima cold seep at atmospheric pressure and sampled gill tissues at 0, 3 and 9 h after collection. Coupled 16S rRNA amplicon sequencing and RNA-Seq were used to follow changes in the gill microbiota, host transcription and descriptive temporal covariation. *Methyloprofundus* remained the dominant bacterial genus throughout the experiment, whereas *Ca. Vesicomyosocius*, the SUP05 cluster, and operational taxonomic units (OTUs) assigned to *Vibrio* and *Pseudoalteromonas* had higher relative abundances at 9 h in the descriptive analysis and exhibited positive temporal covariation. RNA-Seq showed a stronger transcriptional shift at 3 h than at 9 h. Stress-related genes, including HSP70, HSP105, Toll-like receptors, fibrinogen-related proteins, dual oxidase, thioredoxin-system genes, ferritin, taurine transporter and sulfide:quinone oxidoreductase, changed markedly, while energy-consuming ribosome biogenesis was suppressed to conserve energy under combined physical and nutritional stressors. The temporal patterns of *Ca. Vesicomyosocius* and other low-abundance bacterial signals coincided with broad host stress and immune and metabolic transcriptional changes; gene-level microbiome–transcriptome associations were exploratory and did not survive false-discovery-rate (FDR) correction. These results document rapid short-term transcriptional and microbiome changes after recovery, while their causal drivers and consequences require controlled validation.

## Simple Summary

Deep-sea mussels depend on gill bacteria that use methane and sulfur compounds for nutrition. We examined gills of *Gigantidas haimaensis* immediately after recovery from a cold seep and after 3 and 9 h of shipboard maintenance. The methane-using bacterium remained dominant, while several minor sulfur-associated and opportunistic bacterial groups increased in relative abundance over time. Host responses were strongest at 3 h and involved stress, immunity, energy metabolism and protein maintenance. This study provides a cautious description of early post-recovery changes and priorities for future controlled experiments.

## 1. Introduction

Deep-sea chemosynthetic ecosystems, including cold seeps and hydrothermal vents, support dense invertebrate communities through microbial chemosynthesis [1,2]. Bathymodioline mussels are abundant at methane seeps. Their gill bacteriocytes contain intracellular gamma-proteobacterial symbionts capable of methanotrophy, thiotrophy, or both [3,4]. Recovery from the deep sea removes access to native methane and sulfide while simultaneously changing pressure, temperature, oxygen exposure and handling conditions. These co-occurring changes may affect both the host and its symbionts. Methanotrophs obtain carbon and energy by oxidising methane, whereas thiotrophs oxidise reduced sulfur compounds; this metabolic complementarity can broaden the substrates available to the holobiont but may also create dependence on continued chemical supply [4,5].

*Gigantidas haimaensis* (formerly *Bathymodiolus haimaensis*) is the dominant benthic species at the Haima cold seep in the South China Sea, at approximately 1400 m depth [6,7]. Like other deep-sea mussels, *G. haimaensis* harbours a dual gill endosymbiosis, with a dominant methanotroph and a less abundant thiotroph [8,9]. In *G. haimaensis*, *Methyloprofundus* represents the dominant methane-using bacterial partner, whereas *Ca. Vesicomyosocius* represents a less abundant sulfur-associated lineage [8,9]. Within gill bacteriocytes, methanotrophic and thiotrophic symbionts use methane and reduced sulfur compounds, respectively, to support chemosynthetic carbon fixation, while the host provides a protected intracellular habitat. This complementary use of chemical substrates may broaden the metabolic resources available to the holobiont under environmentally variable conditions [2,9,10]. Dual gill symbioses also occur in other bathymodioline mussels and chemosymbiotic invertebrates [11,12,13,14]. Such partnerships are thought to buffer hosts against chemically variable habitats [2].

Genomic and transcriptomic resources for deep-sea chemosymbiotic animals have expanded, but short-term host–symbiont responses to environmental disturbance are still not well understood. Most studies compare populations from different habitats or depths [15,16,17]. Controlled time-series experiments remain uncommon because live deep-sea specimens are difficult to recover and maintain. In *Bathymodiolus* spp., methane starvation can reduce symbiont density [5], and environmental shifts can activate host stress and immune genes [8]. More recent studies in *G. haimaensis* suggest that sampling stress can disrupt host–symbiont interactions within hours after decompression [18], whereas methane limitation can shift endosymbiont metabolism and host tissue remodelling [19]. Building on these studies, the present work focuses specifically on the first 9 h of ex situ shipboard maintenance after recovery and integrates gill 16S rRNA profiling with host RNA-seq within the same short time course. This is particularly important because seabed mining, trawling and climate-linked changes in seep activity can disturb benthic habitats; generate sediment plumes; and alter the chemical, thermal and oxygen conditions experienced by gill symbioses [20,21].

Here, we used 16S rRNA amplicon sequencing and RNA-seq to examine gill tissues collected at 0, 3 and 9 h after recovery of *G. haimaensis* from the Haima cold seep. Specifically, we aimed to (1) characterise temporal changes in gill bacterial relative-abundance patterns and host transcriptional profiles during early ex situ maintenance and (2) explore temporal covariation between these microbial and host molecular patterns. This study provides a time-resolved description of early post-recovery holobiont changes under combined shipboard-maintenance conditions; it was not designed to identify any single causal stressor.

## 2. Materials and Methods

### 2.1. Mussel Collection

Adult *G. haimaensis* specimens were collected from the Haima cold seep in the South China Sea (approximately 1400 m depth) during the HYDZ6-202005 cruise aboard the research vessel (R/V) Haiyang 6 (Wuchang Shipyard, Wuhan, Hubei, China), using the remotely operated vehicle Haima. A study-area map of the approximate Haima cold seep field is provided in Figure S3. Representative post-collection specimens, external morphology and an in situ image of the mussel aggregation at the Haima cold seep are provided in Supplementary Figure S6. After the ROV basket reached the deck, mussels were transferred to insulated containers containing ambient deep-sea water and moved to the shipboard laboratory within 10 min. Three individuals were dissected immediately after transfer to the shipboard laboratory and are referred to here as the post-recovery 0 h group (G0), not as a true in situ baseline. Gill tissues were rapidly dissected, immersed in RNAlater stabilization reagent (Thermo Fisher Scientific, Waltham, MA, USA), and stored at −80 °C until nucleic acid extraction. The remaining mussels were kept in aerated, pre-cooled seawater (10 ± 1 °C) at atmospheric pressure in a dark, climate-controlled chamber. During the 9 h experimental period, the remaining mussels were maintained at a density of 12 individuals per 10 L container in a dark, climate-controlled chamber. They were kept in aerated, pre-cooled, unfiltered surface seawater (approximately pH 8.1, salinity 34.5 psu) at 10 ± 1 °C and atmospheric pressure. Because the experiment was conducted shipboard during an active recovery phase, continuous chemical parameters such as dissolved oxygen and nitrogenous wastes (ammonia, nitrite, nitrate) were not analytically quantified. However, continuous aeration was provided to maintain oxygen saturation, and no water changes were performed during this short timeframe. Crucially, no exogenous methane or sulfide was supplemented to the holding water. Within the 9 h sampling window, the mussels remained closed or exhibited slight gaping without visible severe stress, and no mortality was observed. At 3 h (G3) and 9 h (G9) after capture, three additional individuals were randomly selected at each time point, and gill tissues were dissected and preserved using the same procedure. All dissections were performed under sterile conditions to minimise cross-contamination. This observation is limited to the predefined sampling period and does not demonstrate long-term tolerance to atmospheric-pressure maintenance.

### 2.2. Gill Microbiome Profiling by 16S rRNA Gene Sequencing

Total genomic DNA was isolated from approximately 25 mg of gill tissue using the cetyltrimethylammonium bromide (CTAB)/phenolchloroform method– [22]. DNA quality and concentration were assessed by 1% agarose gel electrophoresis and a NanoDrop 2000 spectrophotometer (Thermo Fisher Scientific). The V3–V4 region of the bacterial 16S rRNA gene was amplified with primers 341F (5′-CCTAYGGGRBGCASCAG-3′) and 806R (5′-GGACTACNNGGGTATCTAAT-3′). Polymerase chain reaction (PCR) was performed in 20 μL reactions containing 4 μL of 5× FastPfu Buffer, 2 μL of 2.5 mM dNTPs, 0.8 μL of each primer (5 μM), 0.4 μL of FastPfu DNA Polymerase (TransGen Biotech, Beijing, China) and 10 ng of template DNA. Cycling conditions were 95 °C for 3 min; 27 cycles of 95 °C for 30 s, 55 °C for 30 s and 72 °C for 45 s; and 72 °C for 10 min. Triplicate PCR products were pooled, gel-purified with the AxyPrep DNA Gel Extraction Kit (Axygen, Corning, NY, USA), quantified, and sequenced on the Illumina MiSeq platform using PE300 chemistry (Illumina, San Diego, CA, USA).

Paired-end reads were demultiplexed, quality-filtered and merged using FLASH (v1.2.7) [23]. Sequences with a Phred score < 20, a length < 200 bp, or ambiguous bases were removed with Trimmomatic (v0.33) [24]. High-quality sequences were clustered into operational taxonomic units (OTUs) at 97% identity using UPARSE (v7.1) [25]. Taxonomy was assigned against the SILVA database (v138) [26] using the Ribosomal Database Project (RDP) classifier (v2.2) [27] with a confidence threshold of 0.7. Singleton OTUs and chloroplast or mitochondrial reads were excluded before downstream analyses. One G0 16S rRNA amplicon library failed to meet the sequencing quality-control criteria and was excluded before downstream analysis. Thus, the final microbiome dataset comprised two G0 samples and three samples each for G3 and G9, all analysed using identical filtering and taxonomic assignment procedures.

### 2.3. RNA Isolation and Transcriptome Sequencing

Total RNA was extracted from approximately 50 mg of gill tissue using TRIzol reagent (Invitrogen, Carlsbad, CA, USA) following the manufacturer’s protocol. RNA integrity was assessed on an Agilent 2100 Bioanalyzer using the RNA 6000 Nano Kit (Agilent Technologies, Santa Clara, CA, USA). Only samples with RNA integrity number (RIN) ≥ 8.0 were used for library preparation. Polyadenylated mRNA was enriched using oligo(dT)-conjugated magnetic beads, fragmented, and used for first-strand cDNA synthesis with random hexamers. Second-strand synthesis was performed with DNA Polymerase I and RNase H. Double-stranded cDNA was end-repaired, A-tailed, ligated to Illumina paired-end adapters, and size-selected for 250–350 bp fragments. Library quality was checked on an Agilent 2100 Bioanalyzer and quantified by quantitative PCR. Sequencing was performed on the Illumina NovaSeq 6000 platform with 2 × 150 bp paired-end reads.

Nine independent RNA-seq libraries were prepared, comprising three biological replicates at each of G0, G3 and G9. All nine libraries passed the pre-sequencing and sequencing quality-control checks and were retained for downstream analysis. Raw reads were processed with fastp (v0.20.0) [28] to remove adapters and low-quality bases using a 4 bp sliding window with an average quality threshold of 15. Clean reads were assembled de novo into transcripts with Trinity (v2.8.5) [29] using default parameters. For each gene, the longest isoform was retained as the representative unigene. Functional annotation was performed by BLASTX searches against the National Center for Biotechnology Information (NCBI) non-redundant protein database (NR) (2.2.28), Swiss-Prot, Pfam, Gene Ontology (GO), Clusters of Orthologous Groups (COG) and Kyoto Encyclopedia of Genes and Genomes (KEGG), with an E-value cutoff of 1 × 10^−5^.

### 2.4. Differential Expression and Functional Enrichment

Gene expression was quantified with RSEM (v1.3.1) [30] following Bowtie2 (v2.2.6) [31] alignment. RSEM-derived expected counts (reported in the analysis output as read counts) were used as the count-based input for differential expression analysis with DESeq2 (v1.20.0) [32]; transcripts per million (TPM) values were used only for expression summaries and visualisation. Differential expression was assessed between G3 and G0 and between G9 and G0. Genes with |log2(fold change)| > 1 and Benjamini–Hochberg-adjusted *p* < 0.05 [33] were defined as differentially expressed genes (DEGs). Volcano plots and heatmaps were generated with ggplot2 and pheatmap. KEGG enrichment was performed with KOBAS [34] using a hypergeometric/Fisher exact test, and *p* values were adjusted by the Benjamini–Hochberg procedure. For the revised KEGG interpretation, only pre-specified categories relevant to translation, energy metabolism, protein homeostasis, DNA maintenance and stress signalling were considered; sensory, synaptic and human-disease labels were not interpreted as gill-specific pathways. Adjusted *p* < 0.05 was considered significant, whereas other displayed categories were treated as exploratory. The study did not include DNA sequencing or a variant-calling workflow; gene mutations were therefore not analysed.

### 2.5. Descriptive Integration of Microbiota and Host Transcriptome

To assess host–microbiome covariation during post-recovery maintenance, Pearson coefficients were calculated between group-mean relative abundances of dominant bacterial genera and group-mean expression of the top 200 variable DEGs. These coefficients were used only to colour the heatmap; no *p*-value or significance threshold was applied. Because only three time-point means entered the calculation and time was not controlled, the analysis is descriptive and cannot test a direct host–microbe association. For the focused analysis, Pearson correlation was calculated between log2(*Ca. Vesicomyosocius* abundance + 1) and log2(fragments per kilobase of transcript per million mapped reads (FPKM) + 1) across the eight available samples; Spearman values were calculated only as an auxiliary sensitivity check. Pearson *p* values from this focused analysis were adjusted across all tested transcripts using the Benjamini–Hochberg procedure.

### 2.6. Statistical Analysis

All statistical analyses were performed in R (v4.0.3). All microbiome analyses were considered exploratory. Alpha-diversity indices were compared among the three time points using Kruskal–Wallis tests, with *p*-values obtained from exhaustive permutations of the 560 possible group-label allocations with the observed group sizes. Bray–Curtis community differences were evaluated by permutational multivariate analysis of variance (PERMANOVA) [35] using the same exhaustive permutation framework. Homogeneity of multivariate dispersion was assessed from distances to group centroids in principal-coordinate space. For the G3-versus-G9 genus-level comparison, the even-depth OTU table was aggregated to genus level. A pseudocount of 0.5 was added, and centred log-ratio (CLR) values were calculated within each sample across the 18 tested genera. Welch’s *t*-tests were performed on these values, and *p*-values were adjusted using the Benjamini–Hochberg procedure; q < 0.05 was considered statistically significant. For transcriptomic analyses, the Benjamini–Hochberg procedure was used to control the false discovery rate. Results are reported as mean ± standard deviation unless otherwise stated.

## 3. Results

### 3.1. Cluster Analysis of Gill Symbiotic Microbial Communities

**Figure 1:**
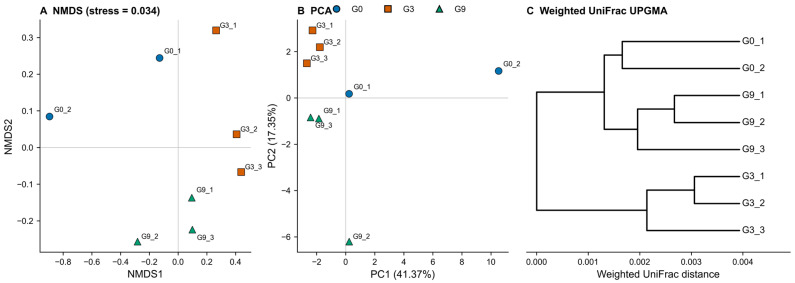
Ordination and clustering of the eight 16S rRNA libraries. (**A**) Non-metric multidimensional scaling (NMDS) based on community dissimilarity (stress = 0.034). (**B**) Principal component analysis (PCA; principal component 1 (PC1) = 41.37% and principal component 2 (PC2) = 17.35% of variance). (**C**) Unweighted pair-group method with arithmetic mean (UPGMA) clustering based on weighted UniFrac distances.

16S rRNA amplicon sequencing generated 564,365 high-quality reads from nine gill libraries, of which eight libraries passed quality control and were used for downstream microbiome analysis (Table S1). Alpha diversity varied across the three sampling times: mean observed richness was 23.0, 12.3 and 18.7 OTUs in G0, G3 and G9, respectively (exploratory permutation Kruskal–Wallis H = 5.44, *p* = 0.0303); mean Shannon indices were 0.255, 0.166 and 0.394 (H = 5.56, *p* = 0.0267); and mean Simpson indices were 0.072, 0.043 and 0.110 (H = 5.62, *p* = 0.0232). In contrast, Faith’s phylogenetic diversity (PD) did not differ detectably among time points (means: 3.051, 1.916 and 2.907; H = 1.81, *p* = 0.5116). Bray–Curtis PERMANOVA indicated an exploratory overall time-point effect (pseudo-F = 11.18, R^2^ = 0.817, *p* = 0.0089), whereas the dispersion test was not significant (F = 0.53, *p* = 0.7647). The non-metric multidimensional scaling (NMDS) representation had low stress (0.034), and the first two principal component analysis (PCA) axes explained 41.37% and 17.35% of variance, respectively (Figure 1). Thus, the ordinations and unweighted pair-group method with arithmetic mean (UPGMA) clustering are reported as descriptive patterns supported by an exploratory global compositional test, while recognizing the small and unequal group sizes. Across all groups, Proteobacteria dominated the microbial community, whereas other phyla accounted for only minor proportions.

### 3.2. Gill Microbial Community Composition

**Figure 2:**
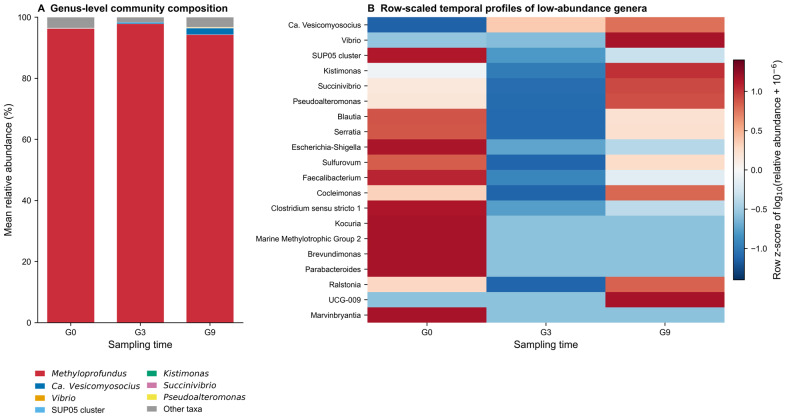
Temporal changes in the gill bacterial community. (**A**) Mean genus-level relative abundance across 0 h (G0), 3 h (G3) and 9 h (G9) post-recovery. (**B**) Row-scaled heatmap of low-abundance genera based on log10 (relative abundance + 10^−6^); colours show the z-score within each genus across the three sampling times.

Taxonomic assignment placed the OTUs in 7 phyla, 9 classes, 22 orders, 24 families and 25 genera. At the genus level, *Methyloprofundus* dominated throughout the experiment, representing 96.3%, 97.8% and 94.3% of relative abundance at G0, G3 and G9 (Figure 2A). Given that only two replicates were available for G0, its baseline community composition is reported cautiously as a descriptive snapshot. Other recurrent genera included *Ca. Vesicomyosocius*, *Vibrio*, the SUP05 cluster, *Kistimonas*, *Succinivibrio* and *Pseudoalteromonas*. Between G3 and G9, the mean relative abundance of *Ca. Vesicomyosocius* changed from 0.51% to 2.15% (4.2-fold), *Vibrio* from 0.002% to 0.16% (69.8-fold), and *Pseudoalteromonas* from 0% to 0.02%. However, none of the 18 genus-level comparisons was significant after Benjamini–Hochberg correction. For the focal taxa, the CLR Welch *p*-values and FDR q-values were 0.2485 and 0.4646 for *Ca. Vesicomyosocius*, 0.2839 and 0.4646 for *Vibrio*, and 0.3439 and 0.5158 for *Pseudoalteromonas*. These values are therefore reported as descriptive relative-abundance patterns rather than statistically confirmed differential abundance. A clade-specific phylogenetic analysis provided broad lineage-level support for the focal OTU assignments; full sequences, reference accessions, methods and trees are provided in Supplementary Methods S1, Tables S4 and S5, and Figure S1.

### 3.3. Genus-Level Microbial Association Patterns

**Figure 3:**
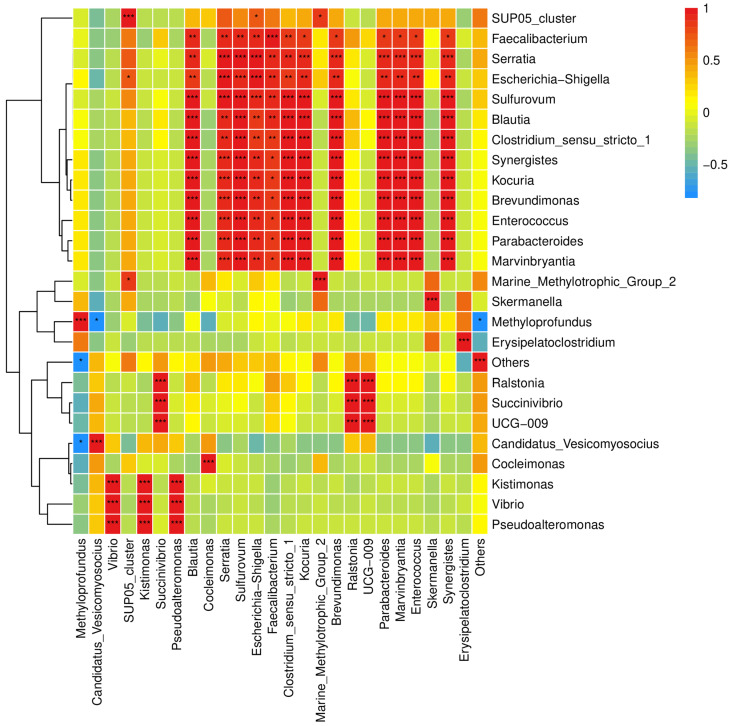
Correlation heatmap of dominant bacterial genera across the time course. Asterisks indicate significant correlations. (*** indicates *p* value < 0.001 ** indicates q value < 0.01 and * indicates q value < 0.05).

Genus-level correlation analysis revealed exploratory co-occurrence patterns within the bacterial community (Figure 3). Several low-abundance assignments showed positive covariation over the time course, including OTUs assigned to *Vibrio* and *Pseudoalteromonas*. These patterns describe compositional covariation only and, in the absence of seawater and blank controls, should not be interpreted as direct microbial interactions, ecological dependencies or confirmed gill colonisation.

### 3.4. Transcriptome Assembly and Differential Expression Overview

RNA-seq generated 58.24 Gb of clean data across nine retained libraries. De novo assembly yielded 97,844 unigenes, with 95.5% complete Benchmarking Universal Single-Copy Orthologs (BUSCOs). Detailed sequencing, assembly and functional-annotation metrics are provided in Tables S2 and S3 and Supplementary Figure S2. More DEGs were detected at 3 h than at 9 h (Supplementary Figure S4). Library-level quality assessment and replicate correlations are provided in Supplementary Figure S2. G3_2 was displaced along PC2 but met all library-quality criteria and was retained; no RNA-seq library was removed as an outlier.

### 3.5. KEGG Pathway Enrichment

**Figure 4:**
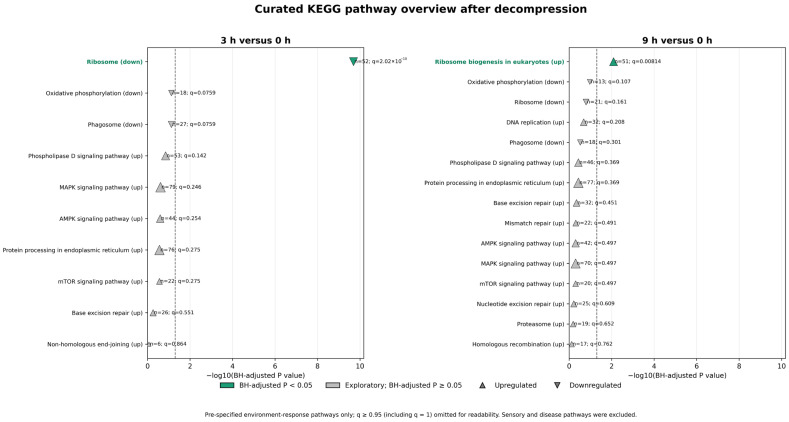
Curated KEGG pathway overview during post-recovery maintenance. Pre-specified environment–response pathways are shown for 3 h vs. 0 h (G3 vs. G0) and 9 h vs. 0 h (G9 vs. G0). Triangles indicate up-regulated (upward) or down-regulated (downward) differentially expressed genes (DEGs) in the manuscript comparison direction; point size reflects gene count. Green indicates Benjamini–Hochberg-adjusted *p* < 0.05; grey indicates exploratory categories with adjusted *p* ≥ 0.05. Pathways with adjusted *p* ≥ 0.95 (including *p* = 1), and sensory or disease pathway labels, were omitted. The vertical dashed line indicates the significance threshold of BH-adjusted q = 0.05.

To limit interpretation of annotation artifacts, KEGG results were re-screened using pre-specified biological categories and Benjamini–Hochberg-adjusted *p* values (Figure 4). At 3 h, ribosome was the only retained significantly enriched pathway and was down-regulated (52 genes; adjusted *p* = 2.02 × 10^−10^). Representative manually curated genes included *RPL21*, *RPL13A*, *RPS14* and *RPL15*. At 9 h, ribosome biogenesis in eukaryotes was significantly enriched among up-regulated genes (51 genes; adjusted *p* = 8.14 × 10^−3^), with representative genes *BMS1*, *UTP12*/*WDR3*, *RIX7*/*NVL* and fibrillarin (*FBL*). Oxidative phosphorylation and phagosome at 3 h, and several signalling, protein-processing and DNA-maintenance categories at 9 h, are displayed as exploratory patterns because they did not meet the adjusted-*p* threshold. Phototransduction, olfactory transduction, G protein-coupled receptor (GPCR)-related synaptic signalling and human-disease pathway labels were excluded from biological interpretation.

### 3.6. Expression Profiles of Key Functional Gene Categories

Several stress- and immunity-related gene categories changed strongly in gill tissues (Table 1). After manual curation, representative molecular chaperones included HSP70 (log2FC = 4.31 at 3 h and 4.16 at 9 h) and HSP105 (log2FC = 2.04 at 3 h and 2.66 at 9 h). A toll-like receptor, fibrinogen-related protein, complement C3 and matrix metalloproteinase were also up-regulated at one or both time points.

Antioxidant-related genes were broadly induced relative to the post-recovery G0 group during ex situ maintenance (Table 2). Dual oxidase 2 showed log2FC values of 3.54 at 3 h and 2.80 at 9 h. Thioredoxin reductase, Cu/Zn superoxide dismutase and ferritin were also up-regulated at one or both time points; peroxidasin was significantly induced at 3 h.

Genes associated with osmolyte transport and sulfide handling also changed over time (Table 3). Taurine transporter was up-regulated at 3 h and 9 h (log2FC = 4.04 and 3.24, respectively). Mitochondrial sulfide:quinone oxidoreductase (SQOR) was also up-regulated (log2FC = 2.89 at 3 h and 3.48 at 9 h).

### 3.7. Exploratory Temporal Covariation Between Microbiota and Host Transcriptome

Supplementary Figure S5 visualises descriptive temporal covariation between group-mean bacterial relative abundance and the top 200 variable host DEGs. Because the heatmap was calculated from three time-point means, it does not provide an inferential test of a direct microbial effect on gene expression.

### 3.8. Exploratory Transcriptional Covariation with the Relative Increase in the Thiotroph Ca. Vesicomyosocius

**Figure 5:**
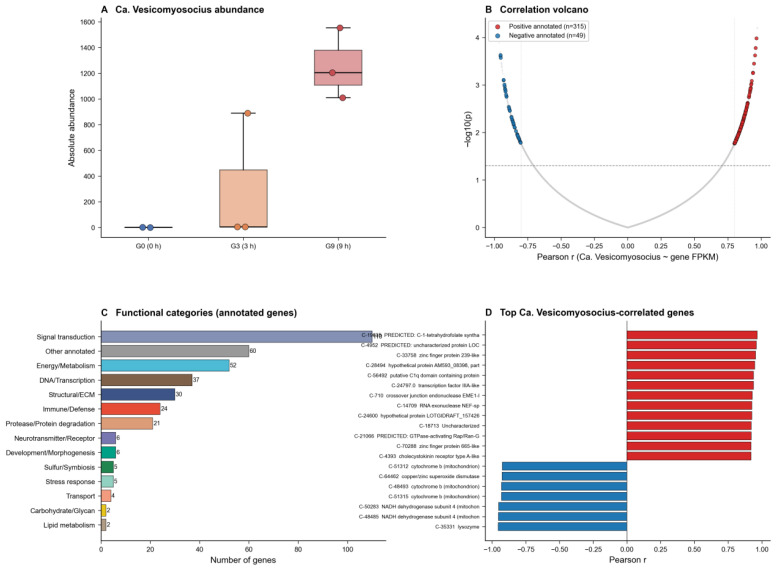
Exploratory host transcriptional covariation with the relative increase in the thiotrophic symbiont *Ca. Vesicomyosocius*. (**A**) Abundance dynamics of *Ca. Vesicomyosocius* across the post-recovery time course. (**B**) Pearson correlation between log2-transformed host gene expression and log2-transformed *Ca. Vesicomyosocius* abundance. In panel B, the horizontal dashed line indicates nominal *p* = 0.05, and the vertical dotted lines indicate |r| = 0.8. (**C**) Functional categories of strongly correlated host genes. (**D**) Top positively and negatively correlated genes. Panels (**C**,**D**) are descriptive displays based on the nominal Pearson screen; no Pearson association remained significant after Benjamini–Hochberg correction across 29,233 transcripts.

Because *Ca. Vesicomyosocius* showed the largest increase among thiotrophic taxa, we performed a focused exploratory correlation analysis between its abundance and host gene expression (Figure 5A,B). A nominal Pearson screen identified 875 annotated transcripts (|r| > 0.8 and unadjusted *p* < 0.05). After Benjamini–Hochberg correction across all 29,233 tested transcripts, no Pearson association remained significant (FDR < 0.05; minimum FDR = 0.476). Accordingly, the functional and gene-level patterns in Figure 5 are presented only as hypothesis-generating descriptive covariation and are not interpreted as evidence of a direct host–symbiont association.

## 4. Discussion

### 4.1. Descriptive Symbiotic Microbiome Changes During Ex Situ Maintenance

During the first 9 h of ex situ maintenance, *Methyloprofundus* remained the dominant bacterial genus. This short-term persistence contrasts with a 6-day low-methane in situ transplantation experiment in *G. haimaensis*, which reported a 30.6% reduction in the methanotrophic symbiont [19]. Long-term laboratory maintenance of the related mussel *Gigantidas platifrons* was likewise accompanied by progressive symbiont loss and later changes in host metabolism, whereas the earliest maintenance period primarily affected symbiont transcription [36]. Together with previous aquarium studies showing changes in symbiont abundance and spatial organisation [5,37], these comparisons suggest that the magnitude of the symbiont response may depend on exposure duration and chemical conditions.

Low-abundance OTUs assigned to *Ca. Vesicomyosocius* and SUP05 exhibited divergent descriptive trajectories during the present time course. In native seep habitats, reduced sulfur compounds can support thiotrophic symbionts, and genomic evidence indicates that *Ca. Vesicomyosocius* possesses the capacity for sulfur oxidation and carbon fixation [2,38]. The relative increase in *Ca. Vesicomyosocius* may motivate an untested hypothesis of transient thiotrophic compensation in response to a putative reduction in methane-derived energy supply. However, the present data do not support this mechanism. Comparable substrate-responsive changes have been documented experimentally in the dual-symbiotic mussel *Bathymodiolus azoricus*. An artificial sulfur pulse increased both the absolute and relative abundance of sulfur-oxidizing symbionts [39], while exposure to reduced-sulfur substrates under pressure produced a rapid and significant increase in their relative abundance [40]. These experiments demonstrate that symbiont composition can respond rapidly to changing chemical conditions, but neither experiment tested compensation for methane-derived energy loss. Because holding-water methane and sulfide concentrations, absolute symbiont abundance and metabolic fluxes were not measured, the present data cannot determine whether such compensation occurred or whether thiotrophic nutrition increased.

Interpretation is further constrained by the taxonomic resolution of the microbiome analysis. The 97% OTU approach supports genus-level patterns but cannot resolve strain-level dynamics, while the short V3–V4 phylogeny establishes only broad affiliation of the focal OTUs. It cannot confirm specific symbiont or strain identities, endosymbiont status or absolute abundance (Figure S1; Supplementary Methods S1; Tables S4 and S5). Accordingly, the descriptive increases in OTUs assigned to *Vibrio* and *Pseudoalteromonas* at G9 should not be interpreted as confirmed opportunistic colonisation. Because the mussels were maintained in unfiltered surface seawater, these low-abundance signals could represent altered gill-associated profiles, seawater-associated taxa, surface-associated cells, handling-related contamination or taxonomic misclassification.

### 4.2. Gill Transcriptome Reprogramming: Oxidative Stress, Immune Activation and Putative Pressure-Related Physiology

Transfer from the Haima cold seep (approximately 1400 m, approximately 14 MPa) to atmospheric pressure reduced hydrostatic pressure by more than two orders of magnitude. Such a shift provides a plausible context for changes in protein folding, membrane fluidity and enzyme kinetics, but these effects were not isolated from the accompanying chemical, thermal, oxygen and handling changes [41,42]. A recent comparison of in situ- and onboard-fixed *G. haimaensis* gills likewise showed that conventional sampling alters both host and symbiont transcript profiles [18]. This conspecific evidence supports interpreting the present redox- and protein-homeostasis-related transcript changes as responses to combined post-recovery conditions rather than to hydrostatic-pressure loss alone. Changes in dissolved oxygen, temperature and chemosynthetic substrates may also promote reactive oxygen species (ROS) production [43]. The induction of antioxidant genes is compatible with combined recovery-related stress, but is not specific evidence of a pressure response. Glutathione peroxidase (GPx), which reduces hydrogen peroxide and lipid hydroperoxides in bivalves [44], increased together with thioredoxin (Trx) and thioredoxin reductase (TrxR). The thioredoxin system is a major redox-regulatory system involved in ROS removal and repair of oxidatively damaged proteins [45,46]. Thioredoxin peroxidase (peroxiredoxin) provides another thiol-dependent route for peroxide reduction [47]. Together, these transcript changes are consistent with activation of cellular redox-homeostasis pathways.

Bivalve molluscs lack adaptive immunity and rely on innate immune mechanisms to recognise microbes and respond to environmental stress [48]. The up-regulation of Toll-like receptors (TLRs), fibrinogen-related proteins (FREPs), C1q-domain-containing proteins (C1qDCs), lysozyme and antimicrobial peptides is consistent with changes in pattern-recognition and effector pathways. In a controlled symbiont-loss and re-contact experiment in *Bathymodiolus platifrons*, pattern-recognition transcripts changed with symbiont status and symbiont re-contact partly reversed immune-associated host responses [49]. This experimental comparison provides context for the immune-related transcripts observed here, but its controlled design differs fundamentally from the combined recovery and shipboard-maintenance conditions examined in the present study. TLRs are a large family of pattern-recognition receptors in mussels that detect pathogen-associated molecular patterns and initiate downstream signalling [48]. The temporal changes in TLR transcripts are consistent with broad stress and microbial-environment changes during ex situ maintenance, but cannot be attributed to any specific bacterial genus. C1qDC proteins, which contain a conserved globular C1q domain with a Gly-X-Y collagen-like motif, can act as pattern-recognition receptors and opsonins that promote phagocytosis in oysters [50]. Several C1qDC transcripts were already highly expressed at G0, showing that these immune-related transcripts were present at the post-recovery baseline; their subsequent changes do not by themselves establish microbial surveillance or damage-control mechanisms.

HSP70 and HSP105, which stabilise and refold denatured proteins, were among the most strongly up-regulated genes (log2FC = 3.77–4.29). Heat-shock proteins are widely used stress biomarkers in marine invertebrates and respond to thermal shock, pressure changes and pollutants [51]. Their induction here may be compatible with general proteotoxic stress during the combined pressure and chemical transition.

Ferritin is an iron-storage protein involved in cellular iron homeostasis and responses to oxidative damage [52]. Metallothioneins are metal-binding proteins involved in metal homeostasis and redox protection [53]. In the present study, the representative ferritin transcript (Cluster-27625.34376) was up-regulated relative to G0 (G3/G0 log2FC = 2.63, FDR = 1.57 × 10^−2^; G9/G0 log2FC = 4.73, FDR = 3.06 × 10^−7^). Together with the induction of redox-defense genes, this pattern is compatible with altered cellular redox and iron-handling responses but is not diagnostic of external iron availability.

Trimethylamine N-oxide (TMAO) stabilises proteins against pressure-induced denaturation and increases with depth in many deep-sea organisms [54,55]. The flavin-containing monooxygenase (FMO)-like transcript associated with TMAO synthesis was down-regulated within 3 h of recovery. This expression pattern is compatible with, but does not demonstrate, altered osmolyte-related physiology after recovery. Because TMAO, taurine and other osmolyte concentrations were not measured, the taurine transporter response is treated only as a candidate hypothesis requiring metabolite validation.

SQOR is a quinone-linked mitochondrial enzyme involved in the sulfide-oxidation pathway [16]. Accordingly, its transcriptional induction is compatible with altered intracellular sulfide turnover and mitochondrial redox handling. In a related deep-sea mussel, host mitochondrial sulfide-oxidation genes responded to experimentally controlled sulfide exposure [8]. In the present experiment, however, external sulfide was neither measured nor supplied. The observed SQOR response may therefore also reflect mitochondrial stress or broader redox imbalance and cannot distinguish among these alternatives. It does not demonstrate increased sulfur-metabolic flux, increased sulfide availability, or thiotrophic compensation.

The nominal gene-level patterns in the focused *Ca. Vesicomyosocius* analysis should be interpreted cautiously. No Pearson association between *Ca. Vesicomyosocius* abundance and host transcript expression survived Benjamini–Hochberg correction across the 29,233 tested transcripts. Thus, the descriptive patterns in Figure 5 are hypothesis-generating only and do not establish host–symbiont coupling or a direct functional effect; interpretation of the host stress response is based on the independent differential-expression results.

### 4.3. Limitations

Several limitations constrain interpretation of this study. G0 is a post-recovery 0 h group rather than a true in situ baseline, and the design did not include pressure-retained or substrate-controlled treatments; pressure loss, temperature and oxygen changes, confinement, handling and substrate deprivation therefore remain confounded. Seawater controls, extraction blanks and PCR-negative-control libraries were unavailable, so low-abundance genera cannot be distinguished confidently from seawater-associated, surface-associated, handling-related or classification-related signals. Finally, microbiome–transcriptome correlations were exploratory, were influenced by time-point structure, and did not establish direct host–microbe interactions. Future histological assessment could provide complementary morphological evidence for interpreting the molecular changes observed during post-recovery maintenance. Gross images, systematic lesion scoring and histological sections were not collected, so morphological tissue changes could not be evaluated.

## 5. Conclusions

1. *Methyloprofundus* remained the dominant gill symbiont during the first 9 h of shipboard maintenance, whereas low-abundance bacterial relative-abundance patterns changed descriptively.
2. Host gill transcriptional responses were strongest at 3 h and involved cellular stress, innate immunity, redox homeostasis and protein maintenance.
3. These results define an early post-recovery holobiont response under combined ex situ conditions, but do not identify individual causal stressors or establish direct host–microbe mechanisms.

## Figures and Tables

**Table 1:** Stress- and innate-immunity-related genes in DEGs.

Functional Category	Representative Unigene	MF Description (Manually Curated)	log2FC (G3/G0)	FDR (G3/G0)	log2FC (G9/G0)	FDR (G9/G0)
HSP70	Cluster-27625.17483	ATP-dependent protein folding chaperone	4.3142	5.20 × 10^−9^	4.1608	3.02 × 10^−6^
HSP105	Cluster-27625.72041	ATP-dependent protein folding chaperone	2.0413	8.95 × 10^−4^	2.6580	1.69 × 10^−3^
Toll-like receptor	Cluster-27625.26600	Pattern-recognition receptor activity	3.3581	1.96 × 10^−2^	--	--
Complement C3	Cluster-27625.13293	Complement component; putative opsonin	2.2861	1.96 × 10^−5^	2.2879	3.35 × 10^−5^
Matrix metalloproteinase	Cluster-27625.18720	Zinc-dependent endopeptidase activity	2.4445	5.08 × 10^−5^	2.6568	3.02 × 10^−5^
Fibrinogen-related protein	Cluster-27625.40877	Fibrinogen C-domain-mediated ligand binding (putative)	2.0742	1.50 × 10^−2^	2.5845	1.02 × 10^−3^

Note: Gene identities and molecular-function (MF) descriptions in Table 1, Table 2 and Table 3 were manually curated using the original NR, Swiss-Prot, Pfam and GO annotation outputs. Values are RNA-seq-derived log2 fold changes and Benjamini–Hochberg-adjusted *p* values (FDR).

**Table 2:** Antioxidant defense genes in DEGs.

Functional Category	Representative Unigene	MF Description (Manually Curated)	log2FC (G3/G0)	FDR (G3/G0)	log2FC (G9/G0)	FDR (G9/G0)
Dual oxidase 2	Cluster-27625.20004	NADPH oxidase activity	3.5390	2.52 × 10^−10^	2.8037	8.76 × 10^−4^
Thioredoxin reductase	Cluster-27625.60625	Thioredoxin-disulfide reductase activity	1.9830	4.35 × 10^−3^	1.9831	1.21 × 10^−3^
Peroxidasin	Cluster-27625.18572	Peroxidase activity	2.3305	5.82 × 10^−3^	--	--
Cu/Zn superoxide dismutase	Cluster-27625.56972	Superoxide dismutase activity	2.2690	6.60 × 10^−3^	2.3482	4.82 × 10^−5^
Ferritin	Cluster-27625.34376	Ferric iron binding	2.6282	1.57 × 10^−2^	4.7294	3.06 × 10^−7^
Thioredoxin-like protein	Cluster-27625.52416	Thiol-disulfide oxidoreductase activity	--	--	1.3297	4.97 × 10^−2^

**Table 3:** Osmolyte-transport and sulfide-handling genes in DEGs.

Functional Category	Representative Unigene	MF Description (Manually Curated)	log2FC (G3/G0)	FDR (G3/G0)	log2FC (G9/G0)	FDR (G9/G0)
Taurine transporter	Cluster-27625.74298	Sodium- and chloride-dependent taurine transporter activity	4.0423	7.52 × 10^−12^	3.2394	2.04 × 10^−13^
Sulfide:quinone oxidoreductase	Cluster-27625.26814	Sulfide:quinone oxidoreductase activity	2.8869	1.18 × 10^−5^	3.4805	1.27 × 10^−4^

## Data Availability

The 16S rRNA amplicon-sequencing and RNA-seq datasets are currently being uploaded to an appropriate public repository. The corresponding accession number(s) will be inserted into the manuscript immediately after they are assigned.

## Supplementary Materials

The following supporting information can be downloaded at: https://www.mdpi.com/article/10.3390/biology15151305/s1, Supplementary Methods S1, Phylogenetic validation of focal OTUs; Table S1, Quality metrics of 16S rRNA gene sequencing; Table S2, Quality metrics of RNA-seq transcriptome sequencing; Table S3, Summary of transcriptome unigene annotation; Table S4, Representative focal OTU sequences; Table S5, NCBI 16S rRNA reference manifest; Figure S1, Clade-specific maximum-likelihood phylogenetic placement of the focal V3–V4 OTUs; Figure S2, RNA-seq library quality control; Figure S3, Study-area map of the Haima cold seep field; Figure S4, Volcano plots of differential expression; Figure S5, Descriptive temporal covariation between dominant bacterial genera and variable host DEGs; Figure S6, Representative specimens, external morphology and in situ aggregation of *G. haimaensis* at the Haima cold seep.
