# Supplementary material for: Rapid Post-Recovery Changes in the Gill Holobiont of the Deep-Sea Mussel Gigantidas haimaensis During Short-Term Ex Situ Maintenance
Source: Biology (Basel). 2026 Aug 5;15(15):1305. doi: 10.3390/biology15151305 (PMC13465936; doi:10.3390/biology15151305)
Supplement: Supplementary file 1 [file biology-15-01305-s001.zip › biology-4409651-supplementary.pdf]

Supplementary Materials

Table S1. Quality metrics of 16S rRNA gene sequencing.

Sample Name	Raw PE	Raw Tags	Clean Tags	Effective Tags	Base	Avg Len	Q20	Q30	GC	Effective (%)
G0.1	110,439	76,728	73,398	68,520	29,312,151	428	95.11	87.43	49.28	62.04
G0.2	101,740	70,915	68,590	65,502	28,040,374	428	95.59	88.41	49.26	64.38
G3.1	112,867	76,470	72,618	67,241	28,810,574	428	95.19	87.68	49.38	59.58
G3.2	105,699	75,687	72,226	66,998	28,700,132	428	94.79	86.78	49.31	63.39
G3.3	98,852	74,496	69,548	62,225	26,656,180	428	94.81	86.82	49.26	62.95
G9.1	100,951	72,904	69,047	63,078	26,994,791	428	94.81	86.74	49.21	62.48
G9.2	99,823	72,456	68,337	61,706	26,401,977	428	95.19	87.46	49.19	61.82
G9.3	103,361	75,528	70,601	61,621	26,384,750	428	95.03	87.2	49.29	59.62

Table S2. Quality metrics of RNA-Seq transcriptome sequencing.

Sample	Raw Reads	Clean Reads	Clean Bases	Error (%)	Q20 (%)	Q30 (%)	GC Content(%)
G0_1	21,678,054	21,062,079	6.32G	0.03	97.85	93.63	34.43
G0_2	22,105,271	21,519,007	6.46G	0.03	97.87	93.73	33.94
G0_3	22,432,448	21,836,978	6.55G	0.03	97.85	93.7	34.19
G3_1	21,775,731	21,176,081	6.35G	0.03	97.96	93.8	35.93
G3_2	21,857,737	21,261,137	6.38G	0.03	97.72	93.37	34.73
G3_3	21,865,783	21,200,522	6.36G	0.03	97.82	93.53	35.82
G9_1	22,554,045	22,070,454	6.62G	0.03	97.69	93.23	35.3
G9_2	23,015,248	22,430,940	6.73G	0.03	97.84	93.67	35.34
G9_3	22,042,163	21,553,320	6.47G	0.03	97.82	93.59	35.61

Table S3. Summary of transcriptome unigene annotation.

Database	Number of Unigenes	Percentage (%)
Annotated in NR	28603	29.23
Annotated in NT	15645	15.98
Annotated in KO	10179	10.40
Annotated in SwissProt	17530	17.91
Annotated in PFAM	26951	27.54
Annotated in GO	26947	27.54
Annotated in KOG	8723	8.91
Annotated in all Databases	2564	2.62
Annotated in at least one Database	45811	46.82
Total Unigenes	97844	100

Supplementary Methods S1. Phylogenetic validation of focal 16S rRNA OTUs

Representative sequences of six focal OTUs (OTU3, OTU4, OTU13, OTU7, OTU12 and OTU8; 429 bp each; Table S4) were analysed in four clade-specific datasets with 38 ingroup references and four outgroup sequences retrieved from NCBI (Table S5). Five reference sequences (MK534991.1, MK534993.1, MK534996.1, MK534997.1 and MK534999.1) were derived from published *G. haimaensis* gill 16S rRNA data (Xu et al., 2019). For each dataset, sequences were aligned with MAFFT using the --auto strategy and trimmed to the 429 aligned positions covered by the focal OTU sequence(s). Maximum-likelihood trees were inferred in IQ-TREE v3.1.3. ModelFinder selected the substitution model according to BIC for each panel: TPM3+G4 (A), TN+F+G4 (B), TN+F+I (C), and HKY+F+I (D). Branch support was evaluated with 1,000 SH-aLRT replicates and 1,000 ultrafast-bootstrap replicates. Trees were rooted with the outgroups listed in Table S5.

OTU3 grouped with vesicomyid-clam-associated thiotrophic references; the comparison included the five published *G. haimaensis* gill 16S references listed above, but did not establish an exact species- or strain-level match. OTU4 and OTU13 were placed within the SUP05/thiotrophic-symbiont assemblage. OTU7 was nearest the *Vibrio fortis* reference cluster, whereas OTU12 was supported only at the *Vibrio* genus level; accordingly, no species-level *Vibrio* assignment is claimed. OTU8 was placed within *Pseudoalteromonas*. These short-region placements are used only to evaluate broad phylogenetic affiliation. They do not independently establish a specific endosymbiont or strain identity, absolute abundance, or proliferation; full-length marker data and/or independent FISH or qPCR validation would be required for those conclusions.

Table S4. Representative 429-bp V3–V4 sequences of the six focal OTUs and their cautious phylogenetic placements.

OTU	Phylogenetic placement used in this revision	Lengt h (bp)	Representative sequence (5′–3′)
OTU3	Vesicomyid-associated thiotrophic lineage (initial *Ca. Vesicomyosocius* assignment)	429	TGGGGAATATTGGACAATGGGCGCAAGCCTGATCCAGCAATACCGCGTGTGTGAAG<br>AAGG<br>CCTGAGGGTTGTAAAGCACTTTCAATTAGGAAGAAGAAATAATGGCTAATATCTATTATT<br>TTTGACGTTACTTTTAGAAGAAGCACCGGCTAACTCCGTGCCAGCAGCCGCGGTAATACG<br>GAGGGTGCAAGCGTTAATCGGAATTACTGGGCGTAAAGCGTGCGTAGGTGGTTTATTAAG<br>TCAGATGTGAAAGCCCTGGGCTCAACCTAGGAACTGCATTTGAAACTGGAAGACTAGAGT<br>ATAGGAGAGGAAAGTGGAATTTCTGGTGTAGCGGTGAAATGCGTAGATATCAGAAGGAAC<br>ATCAATGGCGAAGGCAGCTTTCTGGACTAATACTGACACTGAGGTACGAAAGCGTGGGTA<br>GCAAACAGG
OTU4	SUP05/thiotrophic-symbiont assemblage	429	TGGGGAATATTGGACAATGGGGGCAACCCTGATCCAGCAATGCCGCGTGTGTGAAG<br>AAGG<br>CCTGAGGGTTGTAAAGCACTTTCAATTAGGAAGAAAAATTAATAGTTAATACCTATTAGT<br>CTTGACGTTACTTTTAGAAGAAGCACCGGCTAACTCCGTGCCAGCAGCCGCGGTAATACG<br>GAGGGTGCAAGCGTTAATCGGAATTACTGGGCGTAAAGCGTGCGTAGGCGGTTAATTAAG<br>TCAGATGTGAAAGCCCCGGGCTTAACCTGGGAACTGCATTTGAAACTGGTTAGCTAGAGT<br>ATGGTAGAGGAAAGTAGAATTTCTGGTGTAGCGGTGAAATGCGTAGATATCAGAAAGAAT<br>ACCAATGGCGAAGGCAACTTTCTGGACCAATACTGACGCTGAGGTACGAAAGCGTGGGGA<br>GCAAACAGG
OTU1	SUP05/thiotrophic-symbiont assemblage	429	TGGGGAATATTGGACAATGGGGGCAACCCTGATCCAGCAATACCGCGTGTGTGAAG<br>ACGG

OTU	Phylogenetic placement used in this revision	Length (bp)	Representative sequence (5′–3′)
			CCTGAGGGTTGTAAAGCACTTTCAATTGGGAAGAAAAGCTAGCAGTTAATACCTGTTAGCCTTGACGTTACCTTTAGAAGAAGCACCGGCTAACTCCGTGCCAGCAGCCGCGGTAATACGGAGGGTGCAAGCGTTAATCGGAATTACTGGGCGTAAAGCGTGCGTAGGTGGTTTGTTAAGTCAGATGTGAAAGCCCTGGGCTCAACCTAGGAACTGCATTTGAAACTGGCAAACTAGAGTATAGGAGAGGAAAGTGGAATTTCAGGTGTAGCGGTGAAATGCGTAGATATCTGAAGGAACATCAATGGCGAAGGCAGCTTTCTGGACTAATACTGACACTGAGGTACGAAAGCGTGGGTAGCAAACAGG
OTU7	*Vibrio* genus level; nearest the V. fortis reference cluster	429	TGGGGAATATTGCACAATGGGCGCAAGCCTGATGCAGCCATGCCGCGTGTATGAAGAAGGCCTTCGGGTTGTAAAGTACTTTCAGCAGTGAGGAAGGGGGTGTCGTTAATAGCGGCATCTCTTGACGTTAGCTGCAGAAGAAGCACCGGCTAACTCCGTGCCAGCAGCCGCGGTAATACGGAGGGTGCGAGCGTTAATCGGAATTACTGGGCGTAAAGCGCATGCAGGTGGTTTGTTAAGTCAGATGTGAAAGCCCGGGGCTCAACCTCGGAACTGCATTTGAAACTGGCAAACTAGAGTACTGTAGAGGGGGGTAGAATTTCAGGTGTAGCGGTGAAATGCGTAGAGATCTGAAGGAATACCAGTGGCGAAGGCGGCCCCCTGGACAGATACTGACACTCAGATGCGAAAGCGTGGGGAGCAAACAGG
OTU12	*Vibrio* genus level	429	TGGGGAATATTGCACAATGGGCGCAAGCCTGATGCAGCCATGCCGCGTGTGTGAAGAAGGCCTTCGGGTTGTAAAGCACTTTCAGTCGTGAGGAAGGTAGTGTAGTTAATAGCTGCATTATTTGACGTTAGCGACAGAAGAAGCACCGGCTAACTCCGTGCCAGCAGCCGCGGTAATACGGAGGGTGCGAGCGTTAATCGGAATTACTGGGCGTAAAGCGCATGCAGGTGGTTTGTTAAGTCAGATGTGAAAGCCCGGGGCTCAACCTCGGAATAGCATTTGAAACTGGCAGACTAGAGTACTGTAGAGGGGGGTAGAATTTCAGGTGTAGCGGTGAAATGCGTAGAGATCTGAAGGAATACCGGTGGCGAAGGCGGCCCCCTGGACAGATACTGACACTCAGATGCGAAAGCGTGGGGAGCAAACAGG
OTU8	*Pseudoalteromonas* genus level	429	TGGGGAATATTGCACAATGGGCGCAAGCCTGATGCAGCCATGCCGCGTGTGTGAAGAAGGCCTTCGGGTTGTAAAGCACTTTCAGTAAGGAGGAAAGGTTAAGTGTTAATAGCACTTAGCTGTGACGTTACTTACAGAAGAAGCACCGGCTAACTCCGTGCCAGCAGCCGCGGTAATACGGAGGGTGCGAGCGTTAATCGGAATTACTGGGCGTAAAGCGTACGCAGGCGGTTTGTTAAGCGAGATGTGAAAGCCCCGGGCTCAACCTGGGAACTGCATTTCGAACTGGCAAACTAGAGTGTGATAGAGGGTGGTAGAATTTCAGGTGTAGCGGTGAAATGCGTAGAGATCTGAAGGAATACCGATGGCGAAGGCAGCCACCTGGGTCAACACTGACGCTCATGTACGAAAGCGTGGGGAGCAAACAGG

Note: Taxonomic wording is intentionally conservative because the 429-bp V3–V4 region cannot establish species-, strain-, or endosymbiont-level identity.

Table S5. NCBI 16S rRNA reference sequences used in the four clade-specific phylogenetic analyses.

Panel	Accession / OTU	Role	Length (bp)	NCBI definition or study label	Source relevance
A	OTU_3	this study	429	OTU3 ∣ *Ca. Vesicomyosocius* (this study)	This study
A	MK534991.1	reference	469	MK534991.1 Uncultured Gammaproteobacteria bacterium clone OTU-1 16S ribosomal RNA gene, partial sequence	*G. haimaensis* gill 16S (Xu et al., 2019)
A	MK534993.1	reference	469	MK534993.1 Uncultured Gammaproteobacteria bacterium clone OTU-3 16S ribosomal RNA gene, partial sequence	*G. haimaensis* gill 16S (Xu et al., 2019)
A	MK534996.1	reference	469	MK534996.1 Uncultured Gammaproteobacteria bacterium clone OTU-6 16S ribosomal RNA gene, partial sequence	*G. haimaensis* gill 16S (Xu et al., 2019)
A	MK534997.1	reference	469	MK534997.1 Uncultured Gammaproteobacteria bacterium clone OTU-7 16S ribosomal RNA gene, partial sequence	*G. haimaensis* gill 16S (Xu et al., 2019)
A	MK534999.1	reference	469	MK534999.1 Uncultured Gammaproteobacteria bacterium clone OTU-9 16S ribosomal RNA gene, partial sequence	*G. haimaensis* gill 16S (Xu et al., 2019)
A	PZ315358.2	reference	1499	PZ315358.2 Candidatus Vesicomyidisocius sp. clone C1 16S ribosomal RNA gene, partial sequence	NCBI reference
A	PX530364.1	reference	427	PX530364.1 Candidatus Vesicomyidisocius sp. Atacama clone 1 16S ribosomal RNA gene, partial sequence	NCBI reference
A	JF784423.1	reference	1467	JF784423.1 Bacterium symbiont of Pliocardia sp. SKG-2011 clone AD4503 16S ribosomal RNA gene, partial sequence	NCBI reference
A	MK060679.1	reference	1247	MK060679.1 Phreagena soyoae symbiont isolate T233_11_soyoae 16S ribosomal RNA gene, partial sequence	NCBI reference
A	MK060684.1	reference	1245	MK060684.1 Phreagena soyoae symbiont isolate V1682_A2_21_soyoae 16S ribosomal RNA gene, partial sequence	NCBI reference
A	JX570609.1	outgroup	1362	JX570609.1 Endosymbiont of Ridgeia piscesae clone HF8M2b1 16S ribosomal RNA gene, partial sequence	NCBI outgroup
B	OTU_4	this study	429	OTU4 ∣ SUP05 (this study)	This study
B	OTU_13	this study	429	OTU13 ∣ SUP05 (this study)	This study
B	JX570609.1	reference	1362	JX570609.1 Endosymbiont of Ridgeia piscesae clone HF8M2b1 16S ribosomal RNA gene, partial sequence	NCBI reference
B	JX570606.1	reference	1362	JX570606.1 Endosymbiont of Ridgeia piscesae clone HF8M2b31 16S ribosomal RNA gene, partial sequence	NCBI reference
B	JX570602.1	reference	1371	JX570602.1 Endosymbiont of Ridgeia piscesae clone LF8CM1b5 16S ribosomal RNA gene, partial sequence	NCBI reference
B	JX570599.1	reference	1390	JX570599.1 Endosymbiont of Ridgeia piscesae clone HF8M3b2 16S ribosomal RNA gene, partial sequence	NCBI reference
B	KF780849.1	reference	1214	KF780849.1 Bacterium symbiont of *Bathymodiolus* brevior	NCBI reference

Panel	Accession / OTU	Role	Length (bp)	NCBI definition or study label	Source relevance
		e		clone E2Bathy4 16S ribosomal RNA gene, partial sequence	
B	KF780847.1	reference	1214	KF780847.1 Bacterium symbiont of *Bathymodiolus* brevior clone E2Bathy1 16S ribosomal RNA gene, partial sequence	NCBI reference
B	KF521927.1	reference	1428	KF521927.1 *Bathymodiolus* brevior thioautotrophic gill symbiont clone BBrev16S 16S ribosomal RNA gene, partial sequence	NCBI reference
B	KF521928.1	reference	1428	KF521928.1 *Bathymodiolus* thermophilus thioautotrophic gill symbiont clone BT16S 16S ribosomal RNA gene, partial sequence	NCBI reference
B	KF521930.1	reference	1439	KF521930.1 *Bathymodiolus* marisindicus thioautotrophic gill symbiont clone Bmaris16S 16S ribosomal RNA gene, partial sequence	NCBI reference
B	MW647612.1	reference	1401	MW647612.1 Uncultured Candidatus Thioglobus sp. clone 17574_16_8 16S ribosomal RNA gene, partial sequence	NCBI reference
B	PZ315358.2	outgroup	1499	PZ315358.2 Candidatus Vesicomyidisocius sp. clone C1 16S ribosomal RNA gene, partial sequence	NCBI outgroup
C	OTU_7	this study	429	OTU7 ∣ *Vibrio fortis* (this study)	This study
C	OTU_12	this study	429	OTU12 ∣ *Vibrio* (this study)	This study
C	PZ306311.1	reference	1453	PZ306311.1 *Vibrio fortis* strain ZS4 16S ribosomal RNA gene, partial sequence	NCBI reference
C	PZ306310.1	reference	1438	PZ306310.1 *Vibrio fortis* strain ZS3 16S ribosomal RNA gene, partial sequence	NCBI reference
C	PZ306303.1	reference	1471	PZ306303.1 *Vibrio fortis* strain WS7 16S ribosomal RNA gene, partial sequence	NCBI reference
C	PZ231120.1	reference	1445	PZ231120.1 *Vibrio fortis* strain TA1 16S ribosomal RNA gene, partial sequence	NCBI reference
C	PZ623470.1	reference	1474	PZ623470.1 *Vibrio* alginolyticus strain T3F2S 16S ribosomal RNA gene, partial sequence	NCBI reference
C	PZ623472.1	reference	1474	PZ623472.1 *Vibrio* harveyi strain 1T2F3S 16S ribosomal RNA gene, partial sequence	NCBI reference
C	PZ623482.1	reference	1475	PZ623482.1 *Vibrio* parahaemolyticus strain T4F1G5 16S ribosomal RNA gene, partial sequence	NCBI reference
C	PZ623471.1	reference	1475	PZ623471.1 *Vibrio* fluvialis strain T4F3G5 16S ribosomal RNA gene, partial sequence	NCBI reference
C	PZ623492.1	reference	1466	PZ623492.1 *Vibrio* vulnificus strain WT4F13 16S ribosomal RNA gene, partial sequence	NCBI reference
C	PZ561936.1	outgrou	1412	PZ561936.1 Photobacterium sp. strain CMFRI/DOM/Ph02	NCBI outgroup

Panel	Accession / OTU	Role	Length (bp)	NCBI definition or study label	Source relevance
		p		16S ribosomal RNA gene, partial sequence	
D	OTU_8	this study	429	OTU8｜*Pseudoalteromonas* (this study)	This study
D	OQ626017.1	reference	1364	OQ626017.1 *Pseudoalteromonas* atlantica strain NBRC 103033 16S ribosomal RNA gene, partial sequence	NCBI reference
D	PZ306300.1	reference	1428	PZ306300.1 *Pseudoalteromonas* piscicida strain WS3 16S ribosomal RNA gene, partial sequence	NCBI reference
D	PX915540.1	reference	1456	PX915540.1 *Pseudoalteromonas* rubra strain B1 16S ribosomal RNA gene, partial sequence	NCBI reference
D	OR674766.1	reference	1354	OR674766.1 *Pseudoalteromonas* luteoviolacea strain Z10_BIO 16S ribosomal RNA gene, partial sequence	NCBI reference
D	PZ558852.1	reference	1437	PZ558852.1 *Pseudoalteromonas* sp. strain NHLQS-98 16S ribosomal RNA gene, partial sequence	NCBI reference
D	PZ558850.1	reference	1447	PZ558850.1 *Pseudoalteromonas* sp. strain NHLQS-96 16S ribosomal RNA gene, partial sequence	NCBI reference
D	PZ558824.1	reference	1445	PZ558824.1 *Pseudoalteromonas* sp. strain NHLQS-69 16S ribosomal RNA gene, partial sequence	NCBI reference
D	PZ558800.1	reference	1441	PZ558800.1 *Pseudoalteromonas* sp. strain NHLQS-43 16S ribosomal RNA gene, partial sequence	NCBI reference
D	PZ458719.1	reference	1350	PZ458719.1 Alteromonas mediterranea strain DE 16S ribosomal RNA gene, partial sequence	NCBI reference
D	PZ583805.1	outgroup	1538	PZ583805.1 Alteromonas macleodii strain OP25_84 16S ribosomal RNA gene, partial sequence	NCBI outgroup

Note: Length refers to the downloaded sequence before alignment and trimming. The five MK53499x accessions were generated from *G. haimaensis* gill material and reported by Xu et al. (2019).

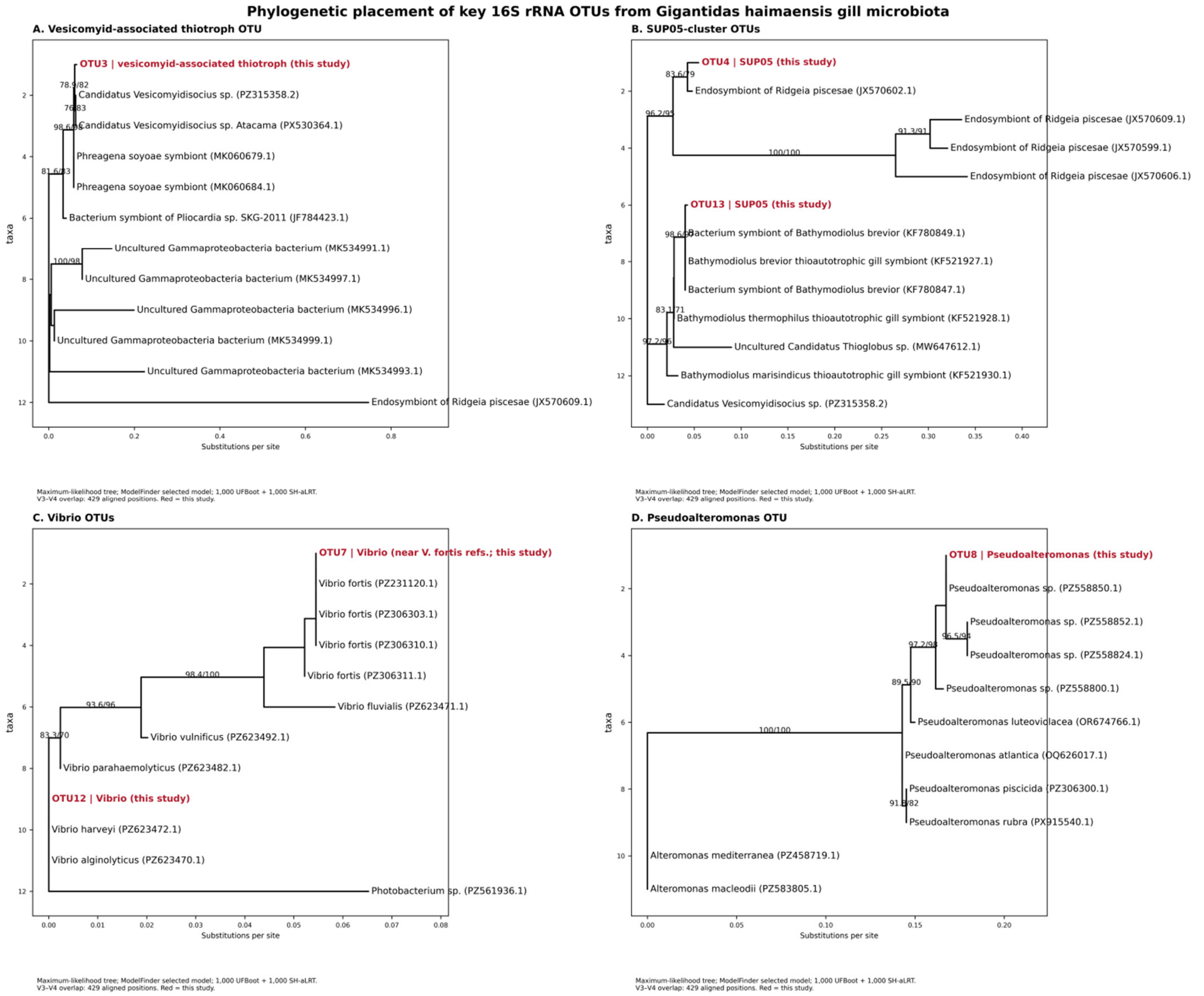

Figure S1. Maximum-likelihood phylogenetic placement of representative 16S rRNA V3–V4 OTUs from *Gigantidas haimaensis* gill microbiota. Four clade-specific trees show (A) vesicomyid-associated thiotroph OTU3, (B) SUP05-cluster OTUs 4 and 13, (C) *Vibrio* OTUs 7 and 12, and (D) *Pseudoalteromonas* OTU8. Red labels indicate OTUs from this study. Branch labels show SH-aLRT support (%) / ultrafast-bootstrap support (%) based on 1,000 replicates each; scale bars indicate substitutions per site. ModelFinder-selected models were TPM3+G4 (A), TN+F+G4 (B), TN+F+I (C), and HKY+F+I (D). The 429-bp V3–V4 placements support broad lineage/genus-level affiliation only and do not establish a specific endosymbiont or strain identity.

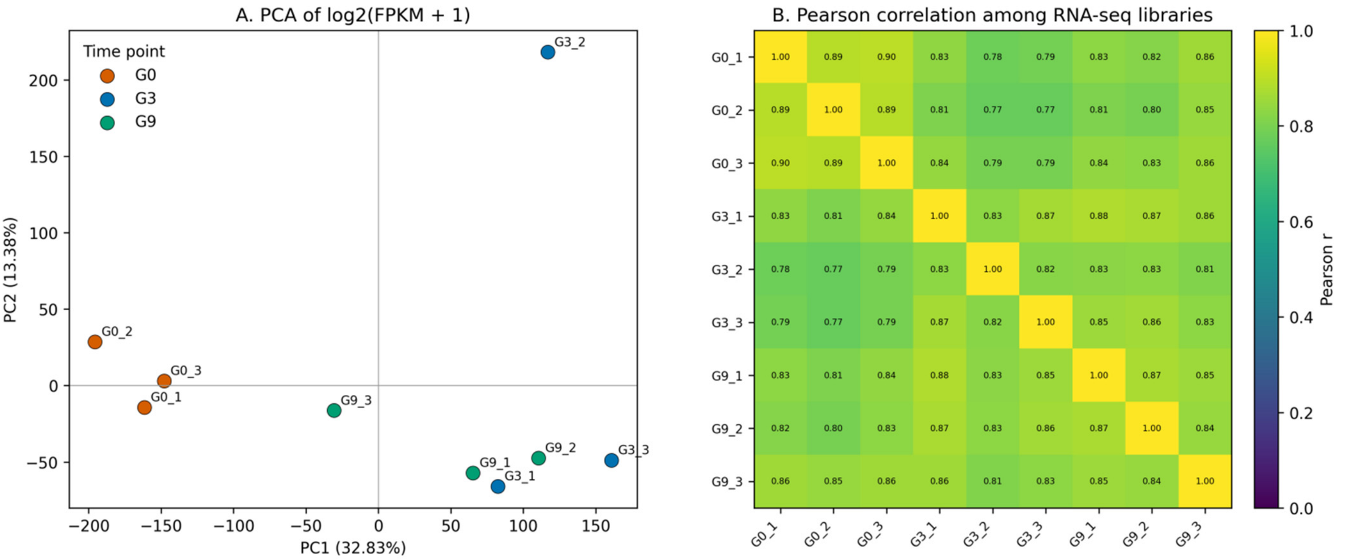

Figure S2. RNA-seq library quality control. (A) PCA of log2(FPKM + 1) values for the nine libraries. (B) Pearson correlation matrix among libraries.

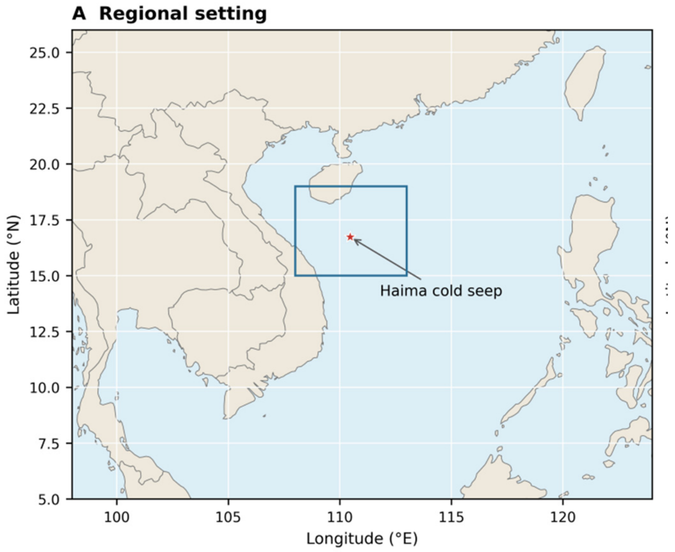

Figure S3. Study-area map of the Haima cold seep field in the South China Sea. (A) Regional setting; (B) sampling-area detail. The star marks the published approximate Haima cold seep field position (110°27.7′ E, 16°43.9′ N; approximately 1390–1400 m). The exact specimen-level ROV station coordinates were not retained in the experimental metadata. Coastline and administrative boundaries were derived from Natural Earth (public-domain data).

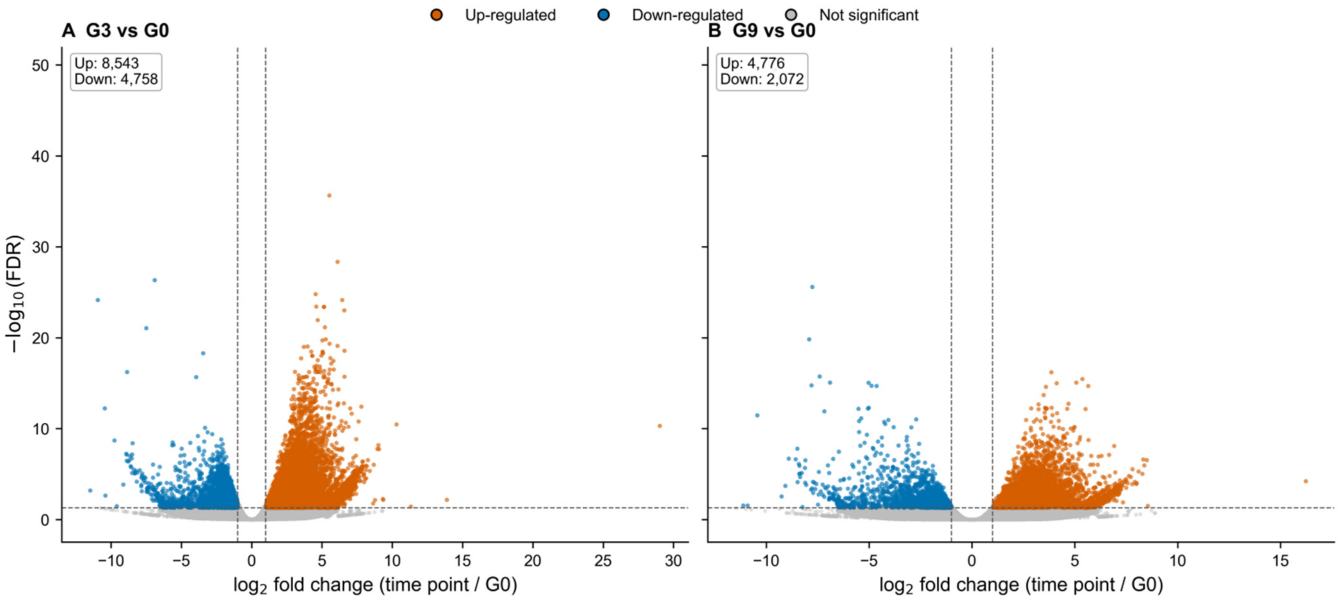

Figure S4. Volcano plots of differential expression in the (A) G3 vs G0 and (B) G9 vs G0 comparisons. Positive log2 fold-change values indicate higher expression at the later time point relative to G0. Differentially expressed genes were defined by |log2 fold change| > 1 and FDR < 0.05.

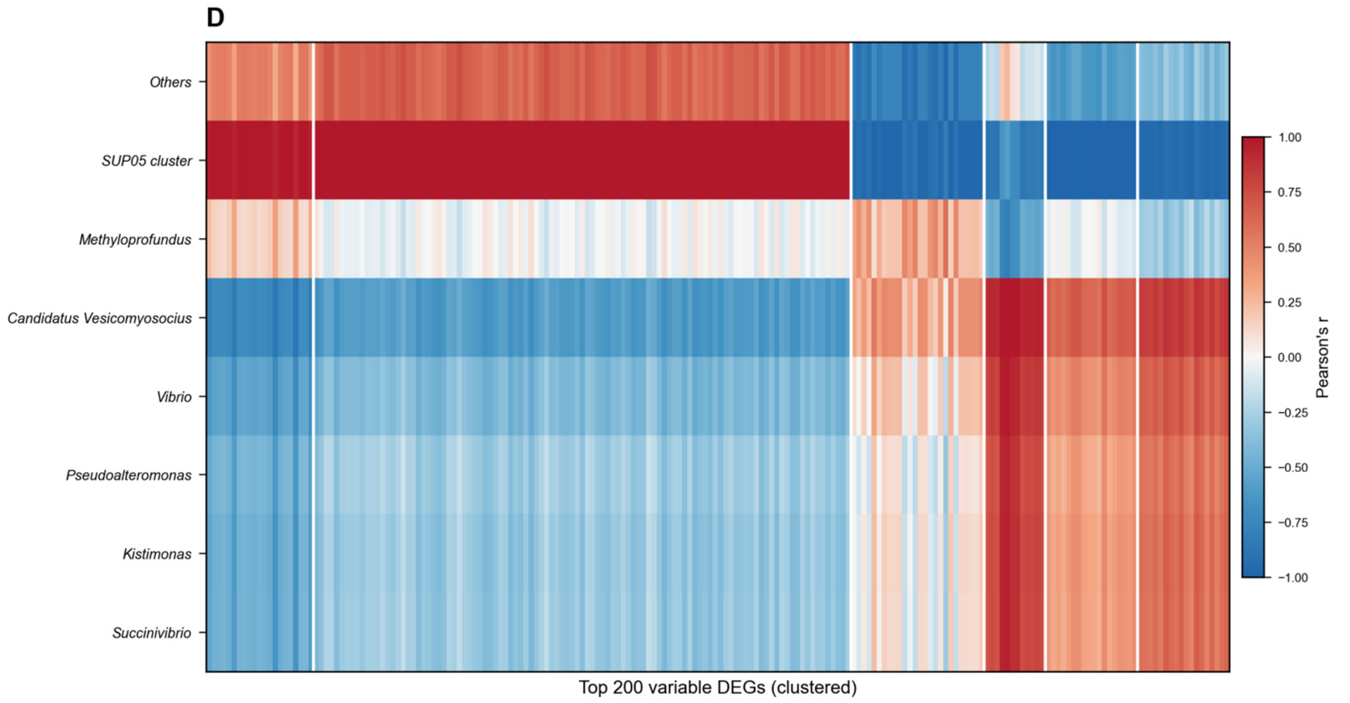

Figure S5. Descriptive heatmap of temporal covariation between dominant bacterial genera and the top 200 variable host DEGs. Pearson coefficients were calculated from the three group-level time-point means and are displayed for visual comparison only; they are not tests of direct host–microbe association.

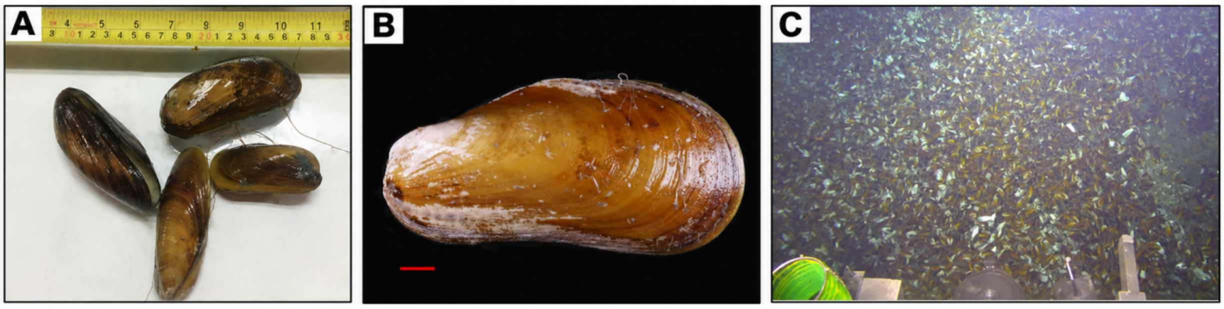

Figure S6. Representative specimens and the in situ aggregation of *Gigantidas haimaensis* at the Haima cold seep. (A) Representative *G. haimaensis* specimens after collection with a ruler for size reference; (B) external morphology of a representative individual (red scale bar = 1 cm); and (C) an in situ image of the dense mussel aggregation at the Haima cold seep.
